# Correlates of Follicular Helper Bias in the CD4 T Cell Response to a Retroviral Antigen

**DOI:** 10.3389/fimmu.2018.01260

**Published:** 2018-06-05

**Authors:** Luca Danelli, Tiziano Donnarumma, George Kassiotis

**Affiliations:** ^1^Retroviral Immunology, The Francis Crick Institute, London, United Kingdom; ^2^Department of Medicine, Faculty of Medicine, Imperial College London, London, United Kingdom

**Keywords:** CD4 T cell response, follicular helper T cells, retroviral infection, CD4 T cell differentiation, TH1 T cells, vaccine vectors and adjuvants

## Abstract

CD4^+^ T cell differentiation is influenced by a plethora of intrinsic and extrinsic factors, providing the immune system with the ability to tailor its response according to specific stimuli. Indeed, different classes of pathogens may induce a distinct balance of CD4^+^ T cell differentiation programmes. Here, we report an uncommonly strong bias toward follicular helper (Tfh) differentiation of CD4^+^ T cells reactive with a retroviral envelope glycoprotein model antigen, presented in its natural context during retroviral infection. Conversely, the response to the same antigen, presented in different immunization regimens, elicited a response typically balanced between Tfh and T helper 1 cells. Comprehensive quantitation of variables known to influence Tfh differentiation revealed the closest correlation with the strength of T cell receptor (TCR) signaling, leading to PD-1 expression, but not with surface TCR downregulation, irrespective of TCR clonotypic avidity. In contrast, strong TCR signaling leading to TCR downregulation and induction of LAG3 expression in high TCR avidity clonotypes restrained CD4^+^ T cell commitment and further differentiation. Finally, stunted Th1 differentiation, correlating with limited IL-2 availability in retroviral infection, provided permissive conditions for Tfh development, suggesting that Tfh differentiation is the default program of envelope-reactive CD4^+^ T cells.

## Introduction

Several divergent and often competing programmes of CD4^+^ T cell differentiation are now well recognized, leading to the development of distinct functional subsets, including T helper (Th) 1, 2, or 17 cells, follicular helper (Tfh) cells, and regulatory T (Treg) cells (1–5). The relative balance of CD4^+^ T cell differentiation to one or more of these functional subsets largely depends on a multitude of T cell-extrinsic factors, with the cytokine milieu naïve T cells encounter during the priming phase playing a major role (1–5). However, CD4^+^ T cell differentiation can also be shaped by T cell-intrinsic properties, such as the relative affinity of the T cell receptor (TCR), favoring development of particular subsets (6–8). The combined effect of such T cell-extrinsic and T cell-intrinsic factors can result in considerable diversity of functional responses, allowing adaptive immunity to modify its response according to the nature of the antigenic stimulus.

Acute viral infections typically induce a CD4^+^ T cell response that is almost exclusively composed of Tfh and Th1 cells, in approximately equal proportion. Indeed, the ratio of Tfh to Th1 cells in the CD4^+^ T cell response to acute lymphocytic choriomeningitis virus (LCMV) has been amply reported close to 1 and 2 for LCMV Armstrong (9–14) and clone 13 (Cl13) (15–19), respectively, and similar results were also reported for influenza A virus infection (20–23).

Several well-defined factors have been demonstrated to influence the balance of Th1 and Tfh cells in response to viruses, as well as other challenges. One of these is the availability of IL-2, determined both by the rate of production by effector CD4^+^ T cells and the rate of consumption by Treg cells or dendritic cells (20, 24–26). IL-2 has been reported to negatively affect Tfh differentiation, in favor of Th1 differentiation (20, 24–26). The balance of Th1 and Tfh differentiations in response to infection with viruses, as well as other classes of pathogens, is also strongly influenced by the nature of the dominant antigen-presenting cell (APC) type (2, 3, 5, 6). For example, antigen presentation by B cells is considered critical for consolidating Tfh differentiation, whereas presentation by macrophages is thought to promote Th1 differentiation (2, 3, 5, 6).

How T cells integrate the multitude of intrinsic and extrinsic factors regulating the balance of their differentiation is not currently completely understood. It is possible that these factors do not operate independently, but are linked at the level of APC-T cell interaction. Indeed, the type of dominant APC determines the cytokine milieu (e.g., IL-12 production or IL-2 consumption) (20, 24–26), the provision of costimulatory signals (e.g., ICOS-L) (5, 27), and the TCR signal strength, given that APC types differ in the potency of stimulation.

We studied the CD4^+^ T cell response to model retroviral antigen from the gp70 envelope glycoprotein of the Friend murine leukemia virus (F-MLV) (28, 29), to assess the relative contribution of individual intrinsic and extrinsic factors, previously linked to the balance of Th1 and Tfh differentiations. Here, we report an atypically strong bias toward Tfh differentiation in response to this retroviral antigen, when presented during natural infection, and describe the variables of the immune response that determine the balance of Th1 and Tfh differentiations in this setting.

## Materials and Methods

### Mice

Inbred C57BL/6 (B6) and CD45.1^+^ congenic B6 (B6.SJL-*Ptprca Pep3b*/BoyJ) mice were originally obtained from The Jackson Laboratory (Bar Harbor, ME, USA). TCRβ-transgenic EF4.1 mice (30), Nur77-GFP transgenic mice (31), B cell receptor-deficient (*Ighm*^−/−^) (32), Tcra-deficient (*Tcra*^−/−^) (33), Rag1-deficient (*Rag1*^−/−^) mice (34), and Rag2-deficient (*Rag2*^−/−^) mice (35) were on the B6 genetic background and were maintained at the Francis Crick Institute’s animal facilities. All animal experiments were approved by the ethical committee of the Francis Crick Institute and conducted according to local guidelines and UK Home Office regulations under the Animals Scientific Procedures Act 1986 (ASPA).

### CD4^+^ T Cell Adoptive Transfer

Single-cell suspensions were prepared from the spleens of donor CD45.1^+^ or CD45.2^+^ TCRβ-transgenic EF4.1 mice or CD45.2^+^ Nur77-GFP EF4.1 doubly transgenic mice, and CD4^+^ T cells were enriched using an immunomagnetic positive selection kit (StemCell Technologies, Vancouver, BC, Canada), at >90% purity. Donor transgenic CD4^+^ T cells (1 × 10^6^ per recipient) were injected into recipient mice intravenously.

### Retroviral Infection and Immunization

The Friend virus (FV) used in this study was a retroviral complex of a replication-competent B-tropic F-MLV (F-MLV-B) and a replication-defective spleen focus-forming virus (SFFV). Stocks were prepared as previously described (36). Mice were injected intravenously with 0.1 mL PBS containing ~50 (low dose), 1,000 (intermediate dose), or 5,000 (high dose) spleen focus-forming units of FV. The F-MLV-NB env_L128I_ variant was generated by mutagenesis of the respective codon (CTC → ATT) in plasmid pLRB302, containing the complete NB-tropic F-MLV clone FB29. The resulting plasmid was then transfected into *Mus dunni* fibroblast cells (*M. dunni* cells; CRL-2017). Stocks of F-MLV-B, F-MLV-NB env_L128I_, or F-MLV-N helper virus were grown in *M. dunni* cells. Mice received an inoculum of ~10^4^ infectious units of helper virus by intravenous injection. Ad5.pIX-gp70 stocks were prepared at a titer of 9 × 10^9^ viral genomes per milliliter by infection of 293A cells as previously described (37). Approximately 5 × 10^8^ Ad5.pIX-gp70 viral genomes per mouse were administered intravenously. Immunization with FBL-3 tumor cells was carried out by intravenous injection of 1.5 × 10^6^ FBL-3 cells (38). For peptide immunization, mice received an intraperitoneal injection of a total of 12.5 nmol of synthetic env_122-141_ peptide mixed in Sigma Adjuvant System (Sigma-Aldrich, St. Louis, MO, USA). Where indicated, recipient mice also received blocking antibodies against PD-1 (10 mg/kg, clone RMP1-14) and LAG3 (10 mg/kg, clone C9B7W) (both from BioXCell, West Lebanon, NH, USA), injected intraperitoneally on days 0, 1, 3, and 5 post FV infection.

### Antibodies and Flow Cytometry

Spleen single-cell suspensions were stained for 20 min at room temperature or at 4°C with directly conjugated antibodies to surface markers. For detection of intracellular antigens, subsequent to surface staining, cells were fixed and permeabilized using the Foxp3/Transcription Factor Staining Buffer Set (Thermo Fisher Scientific, Waltham, MA, USA) according to the manufacturer’s instructions. They were then incubated for 45 min at room temperature with directly conjugated antibodies to intracellular antigens. Zombie UV Fixable Viability Kit (BioLegend, San Diego, CA, USA) was used to label and exclude dead cells from analysis. The following anti-mouse antibodies were used: BV785- or BV711-anti-CD4 (clone GK1.5), PE/Cy7-anti-CD45.1 (clone A20), PE/Cy7-anti-CD279 (PD-1, clone 29F.1A12), BV785-anti-CD150 (SLAM, clone TC15-12F12.2) (from BioLegend); V500-anti-CD44 (clone IM7), BV421- or PerCPCy5.5-anti-CD162 (PSGL1, clone 2PH1), BV421-anti-Ly6C (clone AL-21), PE-anti-Bcl6 (clone K112-91), FITC-anti-Vα2 (clone B20.1) (from BD Biosciences, San Jose, CA, USA); PE-anti-CD25 (clone PC61.5), PE-Cyanine7-anti-CD45R (B220, clone RA3-6B2), APC-eFluor-780-anti-CD45.2 (clone 104), eFluor450-anti-CD45.1 (clone A20), PE-anti-CD223 (LAG3, clone eBioC9B7W), APC-anti-Ter119 (clone TER-119), APC-anti-Vα2 (clone B20.1), FITC- or APC-anti-TCRβ (clone H57-597) (from Thermo Fisher Scientific, Waltham, MA, USA); Alexa(R)488- or Alexa(R)647-anti-TCF1 (clone C63D9) (from Cell Signaling Technology, Danvers, MA, USA). For CXCR5 staining, splenocytes were incubated with biotin rat anti-mouse CXCR5 antibody (clone 2G8, BD Biosciences) at 37°C for 25 min, followed by incubation with APC- or PE-streptavidin (BioLegend) for 20 min at room temperature. FV-infected cells were detected by using surface staining for the glycosylated product of the viral gag gene (Glyco-Gag), using the matrix (MA)-specific monoclonal antibody 34 (mouse IgG2b), followed by an FITC-anti-mouse IgG2b secondary reagent (clone 12-3 from BD). Multi-color cytometry was performed on LSRFortessa flow cytometers (from BD Biosciences) and analyzed with FlowJo v10.1 (Tree Star Inc., Ashland, OR, USA).

### Fluorescence Microscopy

Frozen OCT (Dako)-embedded spleen sections were fixed in cold acetone, stained with fluorescein labeled peanut agglutinin (PNA, Vector Laboratories), and with directly conjugated antibodies against anti-mouse/human B220 (clone RA3-6B2, AlexaFluor 594, BioLegend) and anti-mouse CD45.1 (clone A20, Alexa Fluor 647, BioLegend). Stained sections were mounted in fluorescent mounting medium (Dako) and viewed with an Olympus IX83 inverted microscope system (Olympus Corporation, Shinjuku, Tokyo, Japan).

### Analysis of Single-Cell RNA-Sequencing Data

Gene transcription in env-reactive CD4^+^ T cells was assessed using publicly available single-cell RNA-sequencing data (European Nucleotide Archive accession number PRJEB14043) as previously described (39). These included the transcriptional profiles of single env-reactive donor CD4^+^ T cells isolated from the spleens of wild-type (WT) recipients infected with FV or immunized with Ad5.pIX-gp70, 7 days previously. They also included the transcriptional profiles of single env-reactive donor EF4.1 CD4^+^ T cells that carried a WT *Bcl6* allele (*Bcl6^wt^*) or a conditional *Bcl6* allele (*Bcl6^fl^*), purified from the spleens of WT recipient mice, 7 days after FV infection. Expression values were analyzed using the Qlucore Omics Explorer 3.3 (Qlucore, Lund, Sweden), and pathway analyses were performed using The Database for Annotation, Visualization and Integrated Discovery v6.8 (https://david.ncifcrf.gov/home.jsp).

### Cytokine Gene Transcription and Protein Production

Serum levels of IL-2 were measured on a Luminex system (Bio-Plex 100) using the mouse cytokine kits (Bioplex Mouse cytokine group II and Bioplex Mouse Cytokine Standard; Bio-Rad Laboratories, Hercules, CA, USA) following the manufacturer’s instructions, as previously described (30, 40). Transcription of the indicted cytokine genes in env-reactive CD4^+^ T cells was assessed using publicly available single-cell RNA-sequencing data, as described above.

### Statistical Analyses

Statistical comparisons were made using SigmaPlot 13.0 (Systat Software Inc., Germany) or GraphPad Prism 7 (GraphPad Software, La Jolla, CA, USA). Parametric comparisons of normally distributed values that satisfied the variance criteria were made by unpaired Student’s *t*-tests or One Way Analysis of variance (ANOVA) tests. Data that did not pass the variance test were compared with non-parametric two-tailed Mann–Whitney Rank Sum tests or ANOVA on Ranks tests. *p* Values are indicated by asterisks as follows: *p < 0.05; **p < 0.005; ***p < 0.0005. Hierarchical clustering, principal component analysis, and heatmap production were performed with Qlucore Omics Explorer 3.3 (Qlucore).

## Results

### The CD4^+^ T Cell Response to F-MLV Env Is Heavily Dominated by Tfh Cells

To study CD4^+^ effector T cell development, we employed a well-described adoptive transfer system, where EF4.1 TCRβ-transgenic CD4^+^ T cells, reactive with the dominant H2-A^b^-restricted env_122-141_ epitope within the F-MLV gp70 glycoprotein, were transferred into WT B6 recipients (37, 41). Transferred T cells were primed in recipient mice by infection with FV, a retroviral complex of F-MLV and SFFV that causes chronic infection in B6 mice (28, 29).

**Figure 1:**
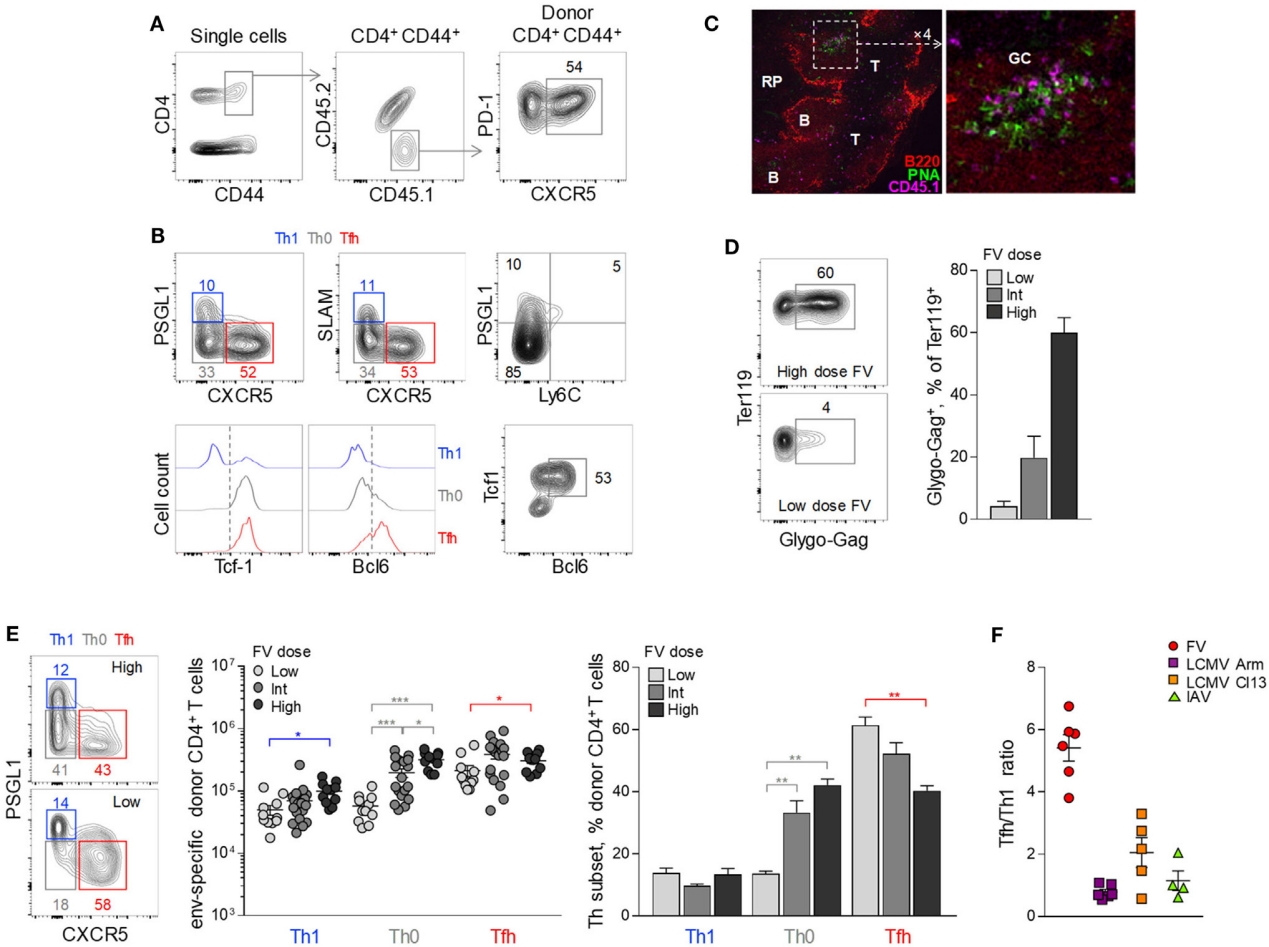
Follicular helper (Tfh) cells heavily dominate the CD4^+^ T cell response to F-MLV env. **(A,B)** CD4^+^ TCRβ-transgenic EF4.1 T cells were adoptively transferred into CD45.1^+^ CD45.2^+^ WT recipients infected with an intermediate dose of Friend virus (FV) (1,000 spleen focus-forming units) and analyzed by flow cytometry in the spleen 7 days later. **(A)** Gating strategy for the identification of env-reactive (CD4^+^ CD44^+^) and Tfh-phenotype (PD-1^+^ CXCR5^+^) donor cells in these recipients. **(B)** Flow cytometric characterization of Th1 (in blue), Th0 (in gray), and Tfh (in red) subsets in env-reactive donor CD4^+^ CD44^+^ T cells. **(C)** CD45.1^+^ CD4^+^ EF4.1 T cells were adoptively transferred into FV-infected CD45.2^+^ WT recipients and detected by immunofluorescence microscopy in spleen sections 7 days later (RP, red pulp; B, B cell zone; T, T cell zone; GC, germinal center). Donor CD4^+^ were identified as CD45.1^+^ cells and germinal centers were visualized by staining with peanut agglutinin (PNA). **(D)** Flow cytometric example of FV-infected Glygo-Gag^+^ Ter119^+^ cells (*left*) and frequency of Glygo-Gag^+^ cells in the Ter119^+^ population (*right*) in the spleens of WT B6 mice infected with different doses of FV. Mean frequency (±SEM, *n* = 3 mice per group) of one representative of two experiments is shown. **(E)** Profile of PSGL1 and CXCR5 expression (*left*), absolute number (*middle*), and mean frequency (±SEM) (*right*) of Th1, Th0, and Tfh cells in env-reactive donor CD4^+^ T cells from the spleen of recipient mice infected with the indicated doses of FV (data are from 4–6 experiments for each FV dose with at least three mice per experiment). **(F)** Tfh/Th1 ratio in env-reactive donor CD4^+^ T cells after FV infection (individual symbols denote the mean Tfh/Th1 ratio in independent experiments with at least 3 mice per experiment), compared the Tfh/Th1 ratio reported in the literature for virus-specific CD4^+^ T cells responding to infection with lymphocytic choriomeningitis virus (LCMV) Armstrong (Arm), LCMV clone 13 (Cl13) or influenza virus PR38 (IAV).

As previously reported (41, 42), a considerable proportion (~50%) of env-reactive EF4.1 CD4^+^ T cells developed a PD-1^high^ CXCR5^+^ phenotype consistent with Tfh cells (Figure 1A). Further detailed phenotypic characterization confirmed the Tfh profile of these cells as CXCR5^+^ PD-1^high^ Bcl6^+^ PSGL1^−^ Ly6C^−^ SLAM^low^ (Figure 1B). Consistent with their phenotype, a large proportion of donor CD4^+^ T cells localized within B cell follicles or germinal centers in the spleens of recipient mice (Figure 1C), further supporting strong Tfh differentiation of env-reactive EF4.1 CD4^+^ T cells.

Notable, however, were the relative paucity (~10%) of env-reactive EF4.1 CD4^+^ T cells with a Th1 phenotype (PSGL1^+^ PD-1^+^ SLAM^+^ CXCR5^−^ Bcl6^−^ Ly6C^−^) (43) and the presence of a sizable population (~35%) of cells that lacked markers of Tfh or Th1 commitment (PSGL1^−^ SLAM^−^ CXCR5^−^) (Figures 1A,B). The latter population, referred here as Th0 to denote their uncommitted state (44), retained TCF-1 expression and expressed low levels of Bcl6 (Figure 1B).

As antigen availability can greatly influence CD4^+^ T cell differentiation, we compared the efficiency of Tfh development in response to different FV loads (Figure 1D). Surprisingly, the proportion of seemingly uncommitted Th0 cells correlated inversely with levels of FV infection, both in terms of absolute numbers and proportion within env-reactive donor CD4^+^ T cells (Figures 1B,E). Indeed, whereas the highest dose of FV primed increased absolute numbers of all three EF4.1 CD4^+^ T cell subsets, Th0 cells exhibited the highest increase (Figure 1E). In contrast, the lowest dose of FV elicited considerable reduced numbers of Th0 and Th1 cells, while favoring development of Tfh cells, which now comprised the overwhelming majority (Figure 1E).

Thus, FV infection induces primarily a Tfh phenotype in env-reactive EF4.1 CD4^+^ T cells and, to a much lesser extent, a Th1 phenotype (Figure 1E), whereas Th2, Th17, Treg, or CTL differentiation is not typically observed (37, 39, 42). This skewing in favor of Tfh differentiation was much more pronounced in FV infection, where there are on average 5.2 times more Tfh than Th1 cells, than in other acute viral infections, where this ratio is consistently reported to be closer to 1 (9–23) (Figure 1F).

### Distinguishable Transcriptional Activity of Uncommitted Th0 Cells

**Figure 2:**
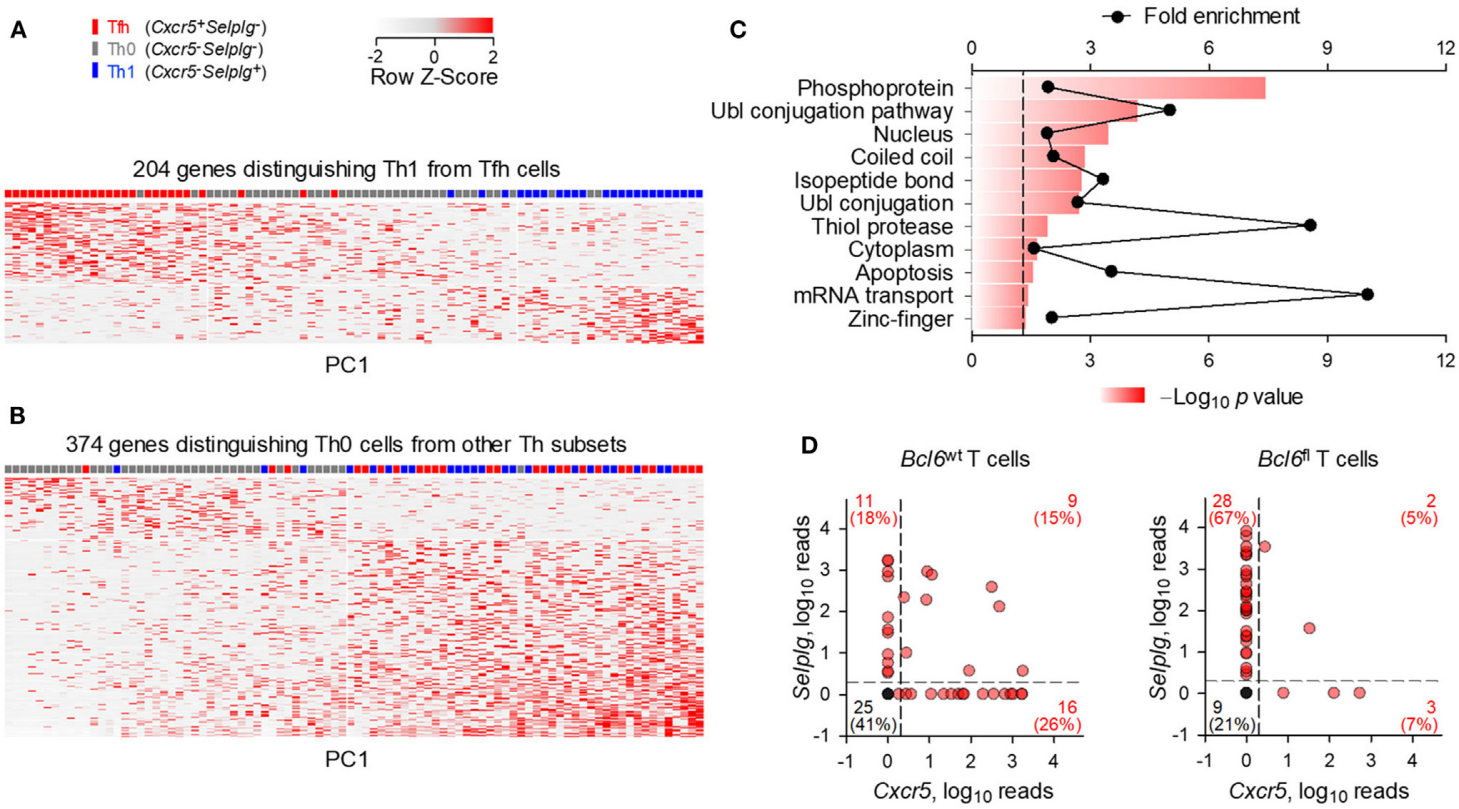
Distinct transcriptional profile of uncommitted Th0 cells. Gene expression previously assessed by single-cell RNA-sequencing of env-reactive donor CD4^+^ T cells isolated from the spleens of WT recipients infected with Friend virus (FV) or immunized with Ad5.pIX-gp70. **(A)** Heatmap of expression values of the 204 most differentially expressed genes (≥2-fold change, *p* ≤ 0.05) between Th1 and follicular helper (Tfh) cells. **(B)** Heatmap of expression values of the 374 most differentially expressed genes (≥2-fold change, *p* ≤ 0.05) between Th0 cells and the other two subsets. Each row and column is an individual gene and cell, respectively, plotted according to their order in principal component analysis. **(C)** Functional pathways revealed by enrichment analysis on genes differentially upregulated (≥2-fold, *p* ≤ 0.05, *q* = 0.05) between Th0 cells and the other two subsets. **(D)** *Cxcr5* and *Selplg* expression in *Bcl6*^wt^ and *Bcl6*^fl^ env-reactive donor EF4.1 CD4^+^ T cells purified from the spleens of recipient mice, 7 days after FV infection. Each red symbol represents an individual cell, whereas single black symbols are plotted for all the cells where *Cxcr5* and *Selplg* expression was undetectable.

The strong bias in Tfh development following FV infection suggested that the seemingly uncommitted Th0 cells were primed env-reactive CD4^+^ T cell that had not successfully completed the program of Tfh differentiation. To better place them in the spectrum of Th differentiation, we compared the transcriptional profiles of Th0 cells using single-cell RNA-sequencing data obtained with env-specific EF4.1 CD4^+^ T cells primed by FV or a replication-defective human adenovirus serotype 5 vector expressing the F-MLV gp70 glycoprotein (Ad5.pIX-gp70) (39). Th1, Tfh, and Th0 cells were defined here according to their expression of *Cxcr5* and *Selplg* (the gene encoding PSGL1). We first selected the top 204 genes, whose expression best differentiated Th1 and Tfh cells (≥2-fold change, *p* ≤ 0.05) (Figure 2A). Transcription of these genes in Th0 cells showed a profile that was intermediate between the Th1 and Tfh extremes (Figure 2A), consistent with their unpolarized phenotype. Direct comparison between Th0 cells and the other two subsets revealed an extensive set of genes that were differentially expressed (≥2-fold change, *p* ≤ 0.05), the majority of which were absent from Th0 cells (Figure 2B; Table S1 in Supplementary Material). Specific to Th0 cells was expression of 88 genes (Table S1 in Supplementary Material), involved in active metabolic pathways (Figure 2C), indicating a highly activated phenotype of Th0 cells, despite incomplete differentiation.

To further probe any subset commitment of Th0 cells, albeit incomplete, we examined their dependency on *Bcl6* expression. This was achieved by using single-cell RNA-sequencing data obtained with env-specific EF4.1 CD4^+^ T cells that carried a WT *Bcl6* allele (*Bcl6^wt^*) or a conditional *Bcl6* allele (*Bcl6^fl^*) that was deleted by Cre-mediated recombination upon T cell activation (39). When primed by FV in WT hosts, 41% (25/61) of *Bcl6^wt^* EF4.1 CD4^+^ T cells displayed a Th0 phenotype (*Cxcr5*^−^*Selplg*^−^), whereas this proportion was reduced to 21% (9/42) in *Bcl6^fl^* EF4.1 CD4^+^ T cells (Figure 2D), despite comparable numerical priming of both types of T cell under these conditions (39). Together, these data suggested that at least a proportion of Th0 cells may have initiated but not completed Tfh differentiation, particularly in conditions of high viral loads.

### Inhibitory Receptors Restrain Full Tfh Maturation in Response to FV Infection

**Figure 3:**
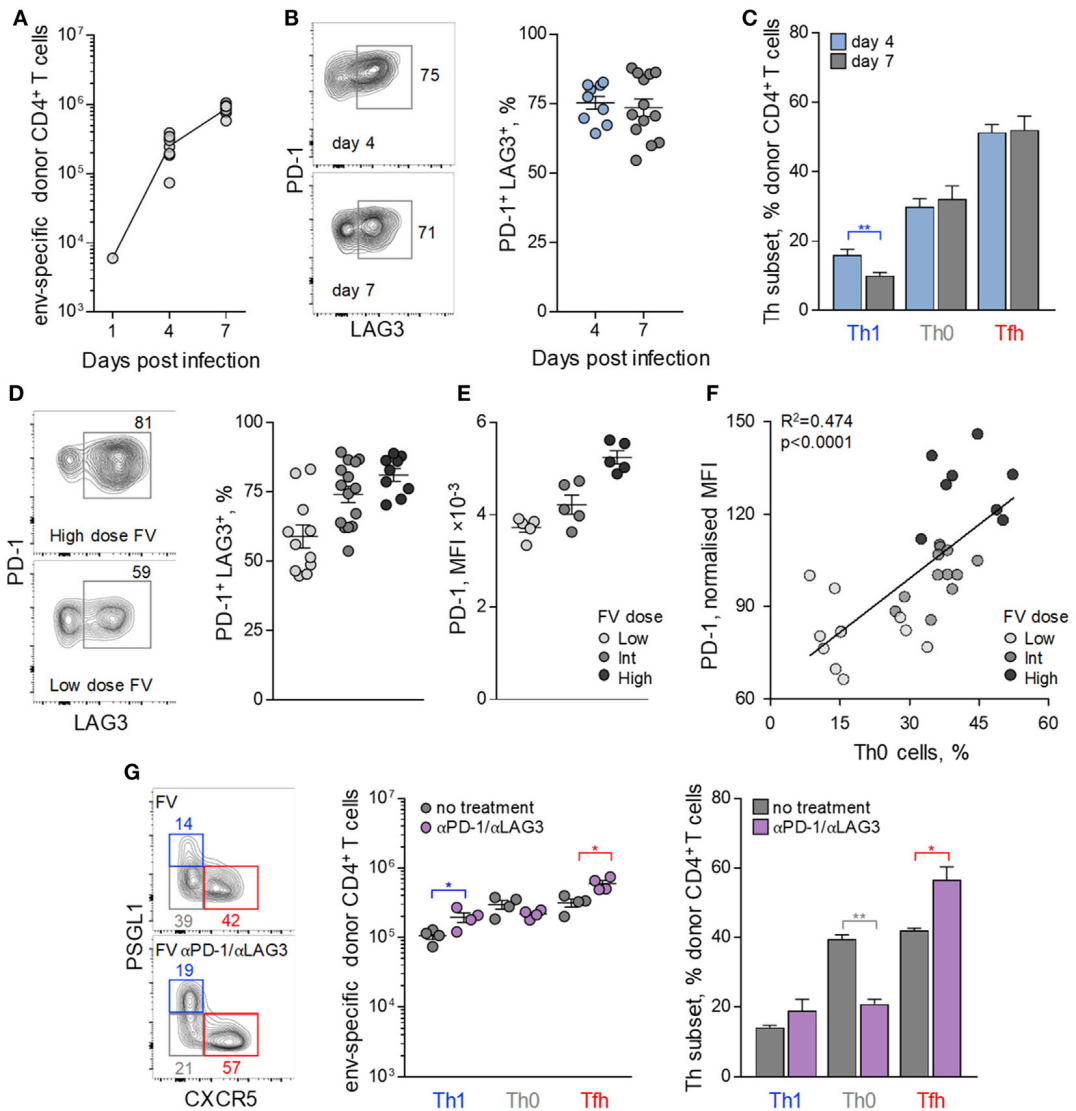
Follicular helper (Tfh) cells maturation control by inhibitory receptor induction. CD4^+^ EF4.1 T cells were adoptively transferred into WT recipients infected with intermediate dose of Friend virus (FV) and analyzed by flow cytometry in the spleens on days 4 (*n* = 9) and 7 (*n* = 13) post-transfer and infection. **(A)** Absolute number of env-reactive CD4^+^ T cells were recovered. **(B)** Flow cytometry profile (*left*) and mean frequency (±SEM) (*right*) of PD-1^+^ LAG3^+^ cells within env-reactive donor CD4^+^ T cells. Each symbol is an individual mouse. **(C)** Mean frequency (±SEM) of T helper (Th) subsets, defined by PSGL1 and CXCR5 expression, in env-reactive donor CD4^+^ T cells. **(D)** Representative flow cytometric profile of PD-1 and LAG3 expression (*left*), mean frequency (±SEM) of PD-1^+^ LAG3^+^ cells, and **(E)** mean fluorescence intensity (MFI) of PD-1 staining in env-reactive CD4^+^ T cells isolated from the spleens of recipient mice on day 7 post transfer and infection with the indicated dose of FV. Each symbol is an individual mouse (*n* = 11, *n* = 14, and *n* = 9 for low, intermediate and high FV dose, respectively). **(F)** Correlation between the frequency of Th0 cells and the MFI of PD-1 expression in env-reactive donor CD4^+^ T cells from the same mice described in **(D)**. **(G)** Flow cytometric analysis (*left*), absolute number (*middle*), and mean frequency (±SEM) (*right*) of Th subsets, defined by PSGL1 and CXCR5 expression, in env-reactive donor CD4^+^ T cells, 7 days after transfer into FV-infected recipients with or without additional treatment with PD-1 and LAG3 blocking antibodies (*n* = 4 from one of two representative experiments).

To further probe how FV loads might influence Tfh differentiation of env-reactive EF4.1 CD4^+^ T cells, we examined the potential effect of inhibitory receptors. At the peak of their response to FV infection, env-reactive EF4.1 CD4^+^ T cells have been previously shown to express high levels of multiple inhibitory receptors, including PD-1 and LAG3 (39). Analysis of the earliest time-points at which donor CD4^+^ T cell expansion can be reliably demonstrated (Figure 3A) revealed that PD-1 and LAG3 expression reached near maximum levels already by day 4 post FV infection (Figure 3B).

Also comparable between days 4 and 7, post infection was the relative ratio of Tfh and Th1 cells in env-reactive EF4.1 CD4^+^ T cells (Figure 3C), in agreement with commitment to Tfh or Th1 differentiation at this early time-point (45). Given that proliferation of donor CD4^+^ T cells is atypically slowing down between days 4 and 7 post FV infection (37), we reasoned that the early induction of inhibitory receptors during FV infection might restrict further differentiation. Consistent with this notion, frequencies of env-reactive EF4.1 CD4^+^ T cells coexpressing PD-1 and LAG3, as well as the intensity of PD-1 expression, directly correlated with both FV loads and the proportion of the uncommitted Th0 subset of env-reactive EF4.1 CD4^+^ T cells (Figures 3D–F).

To test whether the early expression of PD-1 and LAG3 during FV infection was impeding Th differentiation of env-reactive EF4.1 CD4^+^ T cells, we treated recipient mice with PD-1 and LAG3 blocking antibodies. Such treatment during FV infection was previously demonstrated to promote CTL differentiation of donor CD4^+^ T cells, which is not typically observed in FV infection (39). However, the CD4^+^ CTL subset induced by PD-1 and LAG3 blockade during FV infection constituted only ~7% of env-reactive EF4.1 CD4^+^ T cells (39), and it was possible that Tfh differentiation was still favoured. Indeed, PD-1 and LAG3 blockade during FV infection drove efficient differentiation of uncommitted Th0 env-reactive EF4.1 CD4^+^ T cells into Tfh cells, which again formed the large majority, with a small numerical increase in Th1 cells and a small numerical loss of Th0 cells (Figure 3G).

Together, these results suggested that FV infection induces primarily Tfh differentiation of env-reactive EF4.1 CD4^+^ T cells, partly restrained by inhibitory receptor expression, in turn induced by strong TCR signaling.

### Multifactorial Contribution to Tfh Cell Development During FV Infection

The ability of FV infection to promote Tfh differentiation, especially under conditions when PD-1 and LAG3 were not maximally expressed or were blocked, seemed rather exceptional. A host of well-established intrinsic and extrinsic factors may help or hinder Tfh development and, alone or in combination, could account for the dominance of Tfh cells in the response to FV infection. Included among these factors are the avidity of the TCR, the form of antigen and duration of its presentation, the strength of interaction with B cells, and the cytokine environment, particularly the availability of IL-2.

**Figure 4:**
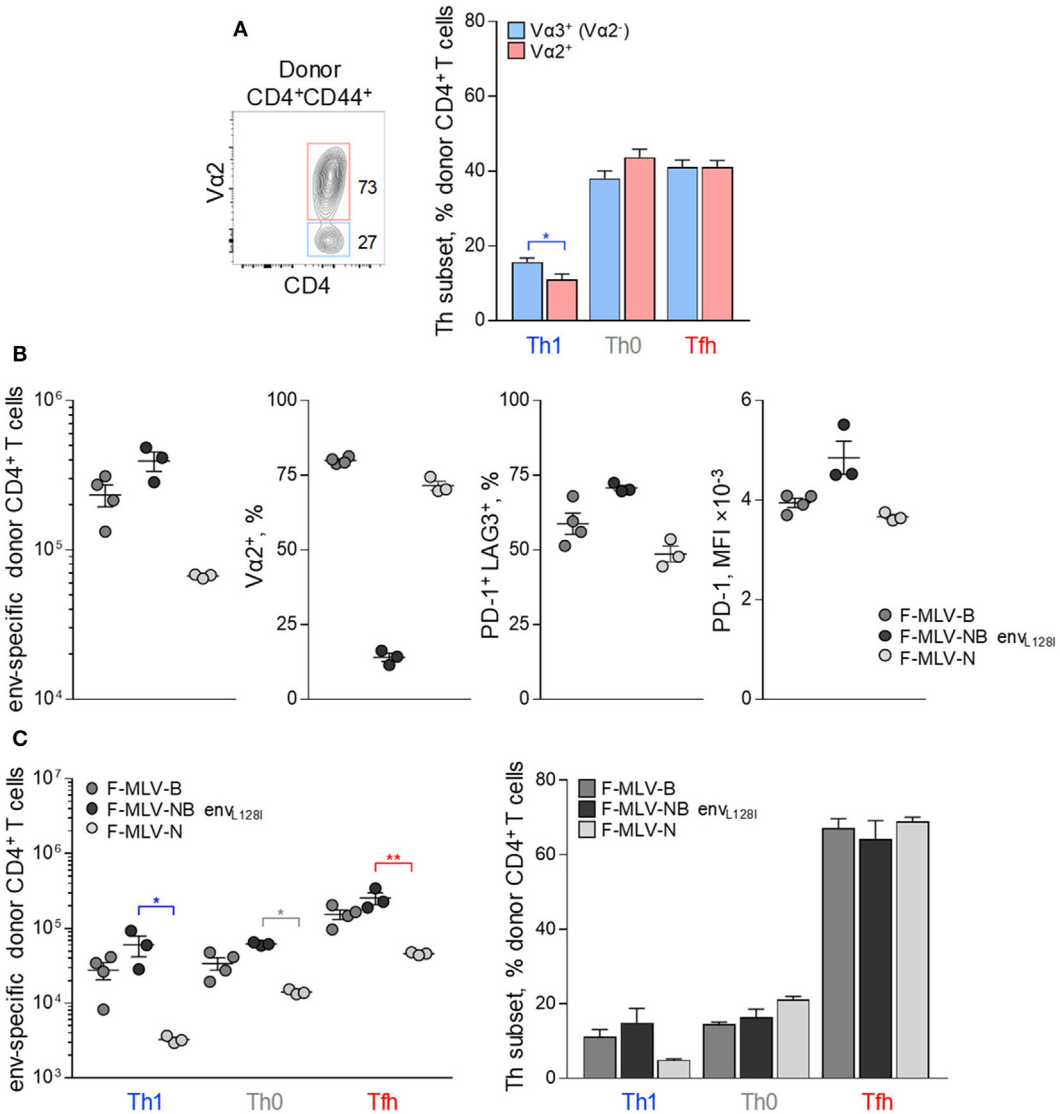
Effect of TCR avidity on the follicular helper cells (Tfh) response to F-MLV env. **(A)** Flow cytometry example of Vα2 expression (*left*) and mean frequency (±SEM) (*right*) of T helper (Th) subsets, defined by PSGL1 and CXCR5 expression, in Vα2^+^ or Vα3^+^ (Vα2^−^) env-reactive donor CD4^+^ T cells, 7 days after transfer into Friend virus (FV) infected recipients (*n* = 10). **(B)** Characterization of clonal expansion and expression of Vα2, PD-1, and LAG3 in env-reactive donor CD4^+^ T cells, 7 days post-transfer in WT recipient mice infected with ~10^4^ infectious units of F-MLV-B, F-MLV-NB env_L128I_, or F-MLV-N. **(C)** Absolute number (*left*) and mean frequency (±SEM) (*right*) of Th subsets, defined by PSGL1 and CXCR5 expression, in env-reactive donor CD4^+^ T cells from the same donor cells as in **(B)**. One representative of two experiments with *n* = 4, *n* = 3, and *n* = 3 mice for F-MLV-B, F-MLV-NB env_L128I_, and F-MLV-N infection, respectively, is shown.

We next investigated how modulation of one or more variables known to affect Tfh cell development, might shape differentiation of CD4^+^ T cells in response to FV. High TCR avidity has long been suggested as a contributor to Tfh cell differentiation in other systems and could contribute to Tfh bias also in response to FV infection (6–8). EF4.1 CD4^+^ T cells comprise a semi-polyclonal repertoire of env-reactive TCRs, differing in their avidity for the cognate antigen, according to the pairing of the transgenic TCRβ chain with endogenous TCRα chains (30, 41). Indeed, clonotypes with higher or lower TCR avidity for H2-A^b^-env_122-141_ can be identified by the use of TCR Vα2 or Vα3 (non-Vα2) endogenous chains, respectively, and used to examine the effect of TCR avidity on Th differentiation (30, 41). Nevertheless, Tfh development was broadly comparable between Vα2^+^ and Vα3^+^ env-reactive CD4^+^ T cell clonotypes, albeit development of Th1 cells appeared more efficient in lower avidity Vα3^+^ clonotypes in response to FV infection (Figure 4A). Thus, the bias in Tfh differentiation of EF4.1 CD4^+^ T cells was observed across clonotypes with a range of TCR avidities.

As an independent way to assess the effect of TCR avidity, we introduced mutations that alter the potency of the F-MLV env_122-141_ epitope to stimulate particular clonotypes. Prior work highlighted the L residue at position 128 as an important contributor to recognition, particularly by higher avidity Vα2^+^ env-reactive CD4^+^ T cell clonotypes (46). Epitopes carrying an L128I mutation behave as strong agonists for all clonotypes (46). F-MLVs expressing variants of the env_122-141_ epitope were also compared with N-tropic F-MLV (F-MLV-N), whose replication in B6 mice is restricted by the product of the *Fv1*^b^ allele, therefore reducing the amount of available antigen.

Friend murine leukemia virus carrying the env_L128I_ epitope induced significantly higher numbers of env-reactive EF4.1 CD4^+^ T cells than WT F-MLV-B, whereas F-MLV-N was less immunogenic (Figure 4B). Consistent with its potency, F-MLV-NB env_L128I_ infection primed Vα2^+^ as well as Vα3^+^ env-reactive CD4^+^ T cell clonotypes, in contrast to WT F-MLV infection, which favored Vα2^+^ clonotypes (Figure 4B). As a result, the frequency of Vα2^+^ clonotypes in env-reactive CD4^+^ T cells was higher following infection with WT F-MLV than with F-MLV-NB env_L128I_ (Figure 4B). Moreover, expression of inhibitory receptors PD-1 and LAG3 was also regulated according to the potency of antigenic stimulation, reaching maximal levels in response to F-MLV env_L128I_ infection (Figure 4B). However, despite notable differences in induced clonal expansion and potency of antigenic stimulation between the mutant F-MLV viruses, differentiation of primed EF4.1 CD4^+^ T cells was highly comparable and still heavily skewed toward the Tfh subset (Figure 4C).

Th differentiation in response to a given antigen is also influenced by the context in which it is presented and in particular by its vector. We, therefore, compared the degree of Tfh differentiation of EF4.1 CD4^+^ T cells induced by F-MLV env_122-141_ in different immunization regimens. These included vaccination with Ad5.pIX-gp70 (47), transient env_124-138_ peptide immunization in Sigma adjuvant and transplantation of the FV-induced FBL-3 tumor cell line (38).

**Figure 5:**
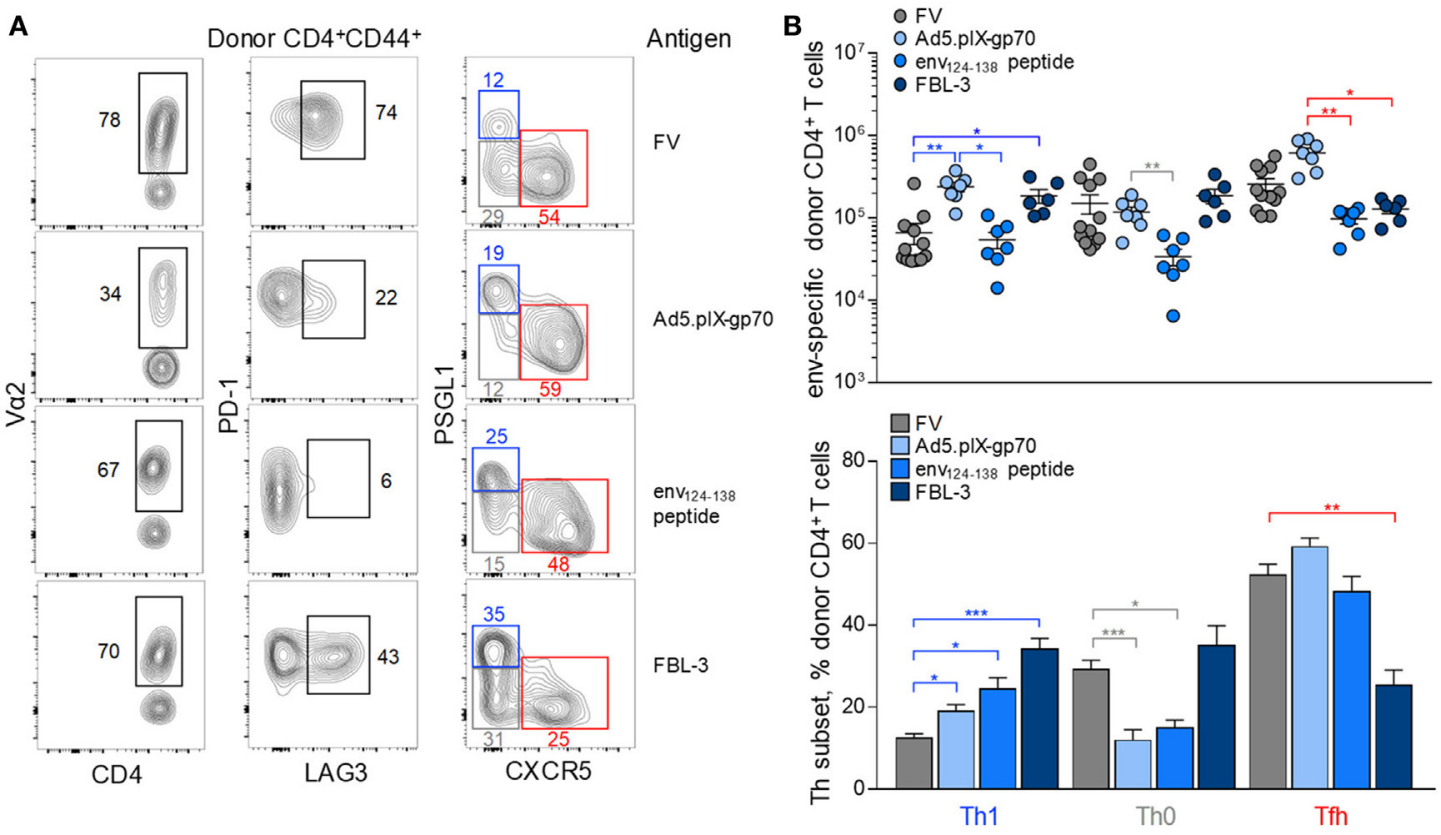
Effect of immunization regimen on the follicular helper cells (Tfh) response to F-MLV env. **(A)** Flow cytometric analysis of Vα2, PD-1, LAG3, PSGL1, and CXCR5 expression in env-reactive CD4^+^ T cells isolated from the spleens of WT recipients on day 7 post transfer and priming with the indicated immunization regimens [intermediate Friend virus (FV) infection, *n* = 3; Ad5.pIX-gp70 vaccination, *n* = 7; env_124-138_ peptide in Sigma Adjuvant System immunization, *n* = 7; FBL-3 challenge, *n* = 6]. **(B)** Absolute number (*top*) and mean frequency (±SEM) (*bottom*) of T helper (Th) subsets, defined by PSGL1 and CXCR5 expression, in env-reactive donor CD4^+^ T cells from the same donor cells as in **(A)**.

Each immunization regimen induced a characteristic pattern of clonotypic composition and inhibitory receptor expression (Figure 5A). In comparison with FV infection, Ad5.pIX-gp70 immunization induced a sizeable Tfh, as well as Th1 population of env-reactive CD4^+^ T cells, as previously shown for CD4^+^ CTL development (39), without concomitant increases in the Th0 subset (Figures 5A,B). Peptide immunization induced lower numbers of env-reactive CD4^+^ T cells, but this reduction affected mostly Th0 and Tfh cells (Figures 5A,B). Finally, FBL-3 tumor cell immunization promoted expansion of Th1 cells, at the expense of Tfh cells, but also primed Th0 cells (Figures 5A,B). Thus, each immunization regimen elicited a somewhat distinct Th differentiation balance in env-reactive EF4.1 CD4^+^ T cells. Nevertheless, Tfh cells continued to be a prominent subset in response to all the regimens (Figure 5B).

**Figure 6:**
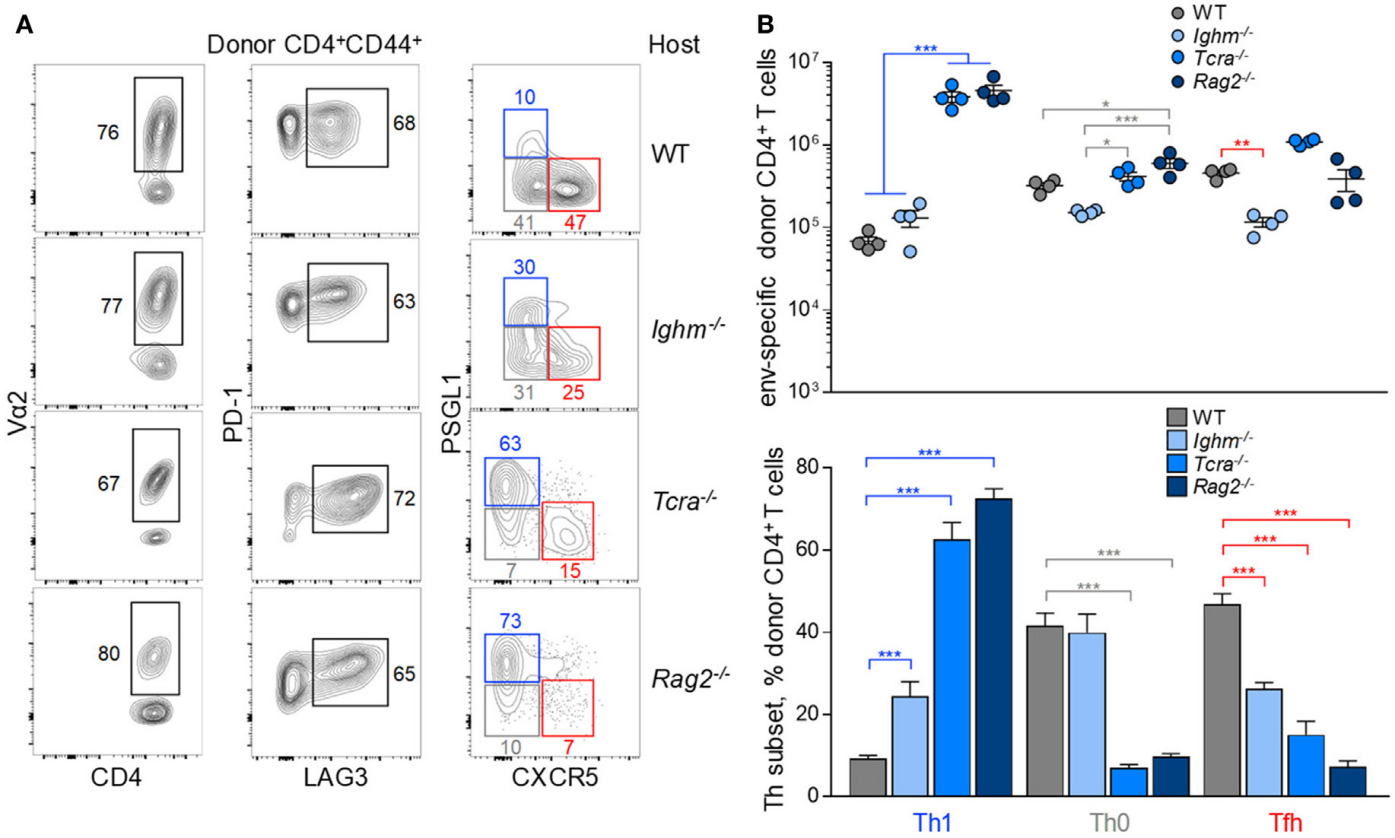
Effect of lymphocyte interaction on the follicular helper (Tfh) response to F-MLV env. **(A)** Flow cytometric analysis of Vα2, PD-1, LAG3, PSGL1, and CXCR5 expression in env-reactive CD4^+^ T cells isolated from the spleens of the indicated recipient mice on day 7 post transfer and intermediate Friend virus (FV) infection (WT host, *n* = 12; *Ighm*^−/−^ host, *n* = 6; *Tcra*^−/−^ host, *n* = 6; *Rag2*^−/−^ host, *n* = 7). **(B)** Absolute number (*top*) and mean frequency (±SEM) (*bottom*) of T helper (Th) subsets, defined by PSGL1 and CXCR5 expression, in env-reactive donor CD4^+^ T cells from the same donor cells as in **(A)**.

Finally, the Tfh differentiation was examined in the context of altered lymphocyte interaction. To this end, EF4.1 CD4^+^ T cells were transferred into FV-infected hosts deficient in B cells (*Ighm*^−/−^), T cells (*Tcra*^−/−^), or both B and T cells (*Rag2*^−/−^). Again, clonotypic composition or inhibitory receptor expression in the env-reactive donor CD4^+^ T cells was characteristic of each type of recipient (Figure 6A). Consistent with the established role for B cells in consolidating Tfh differentiation, env-reactive EF4.1 CD4^+^ T cells produced a larger fraction of Th1 cells in *Ighm*^−/−^ hosts, than in WT hosts (Figures 6A,B). Expectedly, enhanced Th1 differentiation in B cell-deficient hosts was at the expense of Tfh differentiation (Figures 6A,B). Despite this shift, however, Th1-phenotype env-reactive EF4.1 CD4^+^ T cells in *Ighm*^−/−^ hosts did not outnumber those with a Tfh phenotype or those with an uncommitted Th0 phenotype, with all three subsets represented in almost equal proportions (Figure 6B). Surprisingly, in comparison with WT or *Ighm*^−/−^ hosts, Th1 differentiation of env-reactive EF4.1 CD4^+^ T cells was significantly more pronounced in *Tcra*^−/−^ hosts, where Th1 cells now became the dominant subset (Figures 6A,B). Skewed differentiation was even more pronounced in *Rag2*^−/−^ hosts, where env-reactive EF4.1 CD4^+^ T cells developed almost exclusively (~75%) into Th1 cells (Figures 6A,B). This shift in favor of Th1 differentiation in *Tcra*^−/−^ and *Rag2*^−/−^ hosts was driven by approximately 60-fold higher expansion of Th1 cells in such T cell-lymphopenic hosts, in comparison with T cell-replete hosts.

Together, these results highlighted the multitude of intrinsic and extrinsic factors that influence the balance of Tfh and Th1 differentiation in response to F-MLV env, but also indicated that each of these factors may contribute to a different degree, with T cell lymphopenia exerting the strongest influence.

### Reduced IL-2 Availability During FV Infection Facilitates Tfh Development

In addition to increased availability of antigenic peptide-MHC complexes, a well-described effect of T cell lymphopenia is reduced T cell competition for other growth signals, such as cytokines. Consumption of effector CD4^+^ T cell-produced IL-2 by Treg cells or dendritic cells has been shown to promote Tfh differentiation, at the expense of Th1 differentiation in a variety of experimental systems (20, 24–26). It was, therefore, possible that the strong bias toward Tfh differentiation in FV infection was due to defective IL-2 signaling, either due to lack of production or to increased consumption by cells other than effector CD4^+^ T cells.

**Figure 7:**
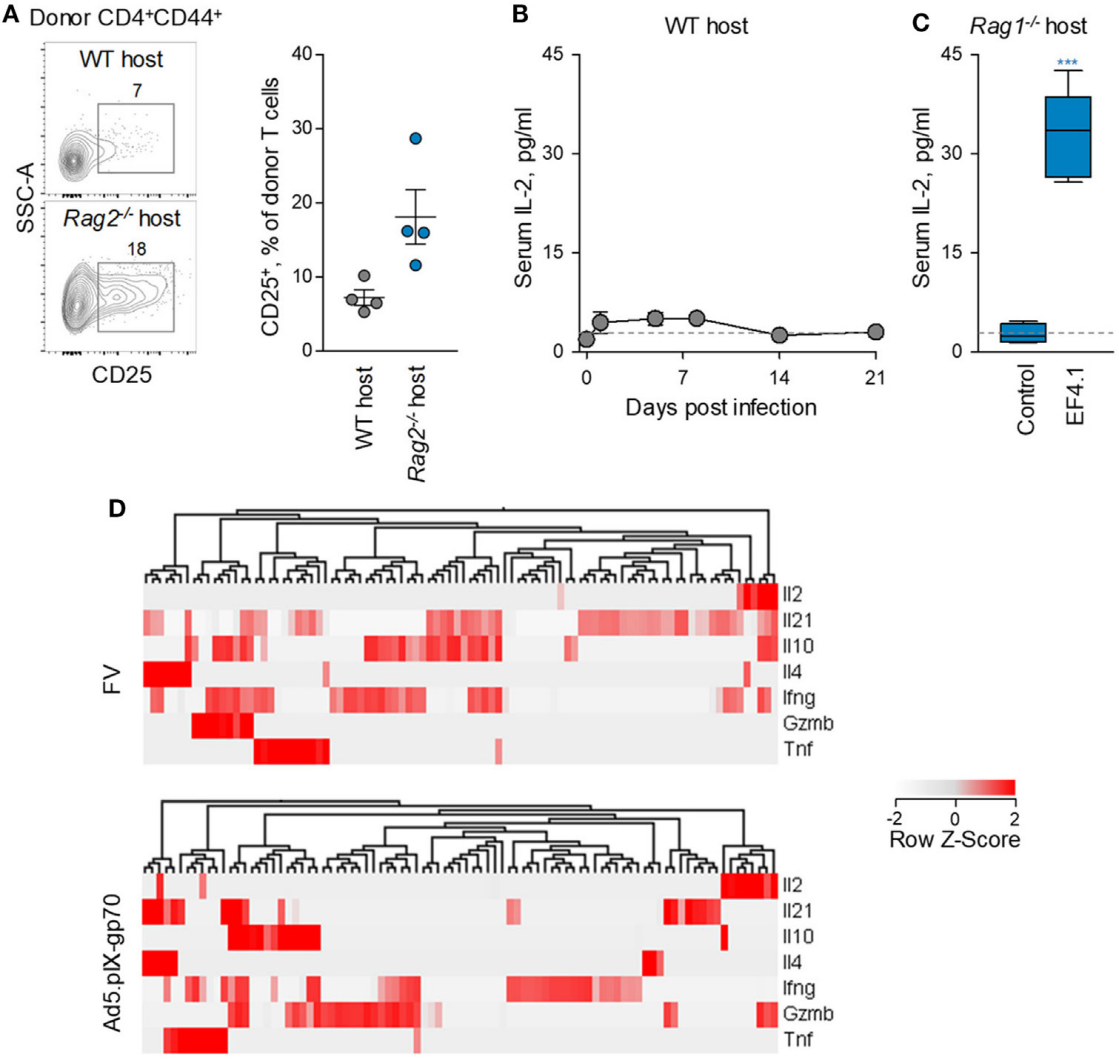
Defective IL-2–IL-2R axis in CD4^+^ T cell response to F-MLV env. **(A)** Flow cytometric example (*left*) and frequency of CD25 expressing cells (*right*) expression in env-reactive donor CD4^+^ T cells, 7 days after transfer into Friend virus (FV) infected WT or *Rag2*^−/−^ recipients. **(B)** Mean (±SEM) levels of IL-2 in the serum of WT B6.*Fv2^s^* mice at different time-points after intermediate FV infection (*n* = 4–5 per time-point). **(C)** Mean (±SEM) levels of IL-2 in the serum of *Rag1*^−/−^ mice 35 days after infection with an intermediate dose of FV (control, *n* = 4) and those additionally received CD4^+^ EF4.1 T cells at the time of infection (EF4.1, *n* = 5). **(D)** Heatmap and hierarchical clustering of cytokine gene expression, assessed by single-cell RNA sequencing, in env-reactive donor CD4^+^ T cells isolated from the spleens of WT recipients infected with FV or immunized with Ad5.pIX-gp70. Each column represents an individual cell.

Consistent with this hypothesis, expression levels of CD25 were lower when env-specific EF4.1 CD4^+^ T cells were primed in WT than in or *Rag2*^−/−^ hosts (Figure 7A), although most env-reactive donor CD4^+^ T cells did not exhibit detectable CD25 expression in either type of host. As expression of CD25 may also be induced by IL-2 signaling, it was possible that, as well as reduced responsiveness of EF4.1 CD4^+^ T cells to IL-2, production or availability of IL-2 was reduced in WT hosts. Indeed, serum IL-2 was minimally detected during FV infection of lymphocyte-replete hosts, even when B6 mice with increased genetic susceptibility, provided by the *Fv2*^s^ allele (36), were used (Figure 7B). In contrast, transfer of EF4.1 CD4^+^ T cells into FV-infected lymphocyte-deficient *Rag1*^−/−^ hosts led to readily detectable serum IL-2 (Figure 7C).

IL-2 transcription was previously found to be reduced in env-specific EF4.1 CD4^+^ T cells responding to FV infection than to Ad5.pIX-gp70 immunization (37). However, relative reduction of IL-2 transcription in FV infection could simply reflect the strong skewing toward Tfh cells, which may not produce IL-2. To further investigate the nature of IL-2-producing CD4^+^ T cells, we searched for correlates of IL-2 production in single-cell RNA-sequencing data obtained with env-specific EF4.1 CD4^+^ T cells primed by FV or Ad5.pIX-gp70 (39). This analysis revealed that most EF4.1 CD4^+^ T cells transcribing *Il2* in response to FV infection, also transcribed *Il21, Il10* or *Ifng* (Figure 7D). In contrast, transcription of distinct cytokine genes was largely restricted to different EF4.1 CD4^+^ T cells responding to Ad5.pIX-gp70 immunization, with most *Il2*-positive cells lacking transcripts for other cytokines, with the exception of *Gzmb* (Figure 7D). These data suggest that env-specific EF4.1 CD4^+^ T cells producing IL-2 following FV infection or Ad5.pIX-gp70 immunization display disparate functional properties. Together, these results highlighted the important contribution of reduced IL-2 production and availability to the dominant Tfh skewing in the CD4^+^ T cell response to FV infection.

### Distinct Correlates of Tfh and Th1 Differentiation in Response to FV Infection

**Figure 8:**
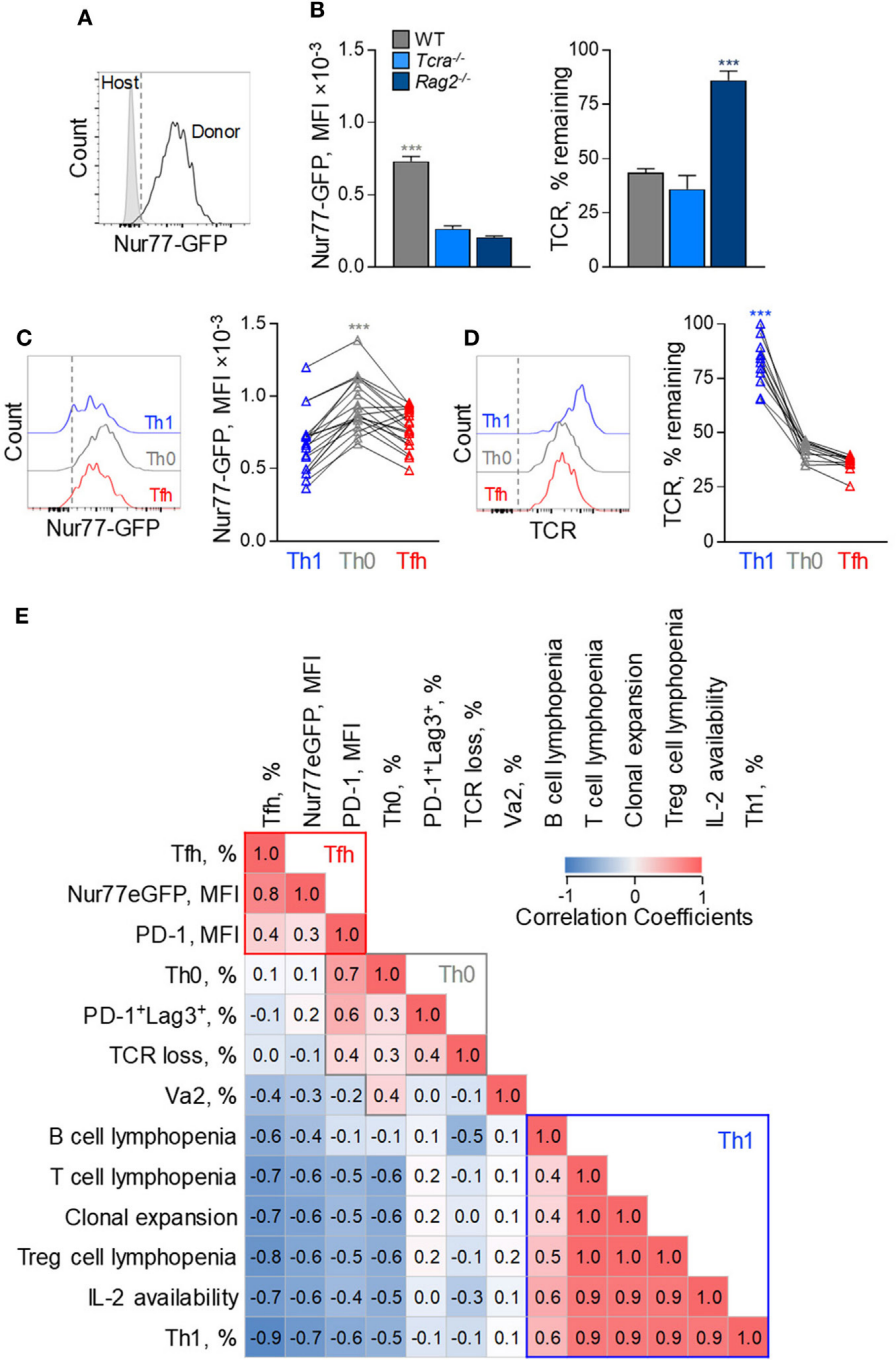
Correlates of follicular helper cells (Tfh) and Th1 CD4^+^ T cell response to F-MLV env. **(A)** Representative flow cytometric profile of GFP expression in Nur77-GFP EF4.1 doubly transgenic env-reactive donor CD4^+^ T cells, 7 days post transfer into Friend virus (FV)-infected WT recipients. **(B)** Mean fluorescence intensity (MFI) (±SEM) of Nur77-GFP expression (*left*) and levels of TCRβ expression (*right*) in env-reactive donor CD4^+^ T cells, 7 days post transfer into WT (*n* = 4), *Tcra*^−/−^ (*n* = 3) or *Rag2*^−/−^ (*n* = 4) recipients, infected with an intermediate dose of FV. One representative of three experiments is shown. **(C)** Flow cytometric profile (*left*) and MFI of Nur77-GFP expression (*right*) and **(D)** flow cytometric profile (*left*) and levels of TCRβ expression (*right*) in T helper (Th) subsets, defined by PSGL1 and CXCR5 expression, in env-reactive donor CD4^+^ T cells, 7 days after transfer into FV-infected WT recipients (*n* = 19). Lines connect values from individual recipients. **(E)** Matrix of correlation coefficients between the indicated variables and Th subset differentiation.

To investigate possible correlates of Tfh bias in FV infection, we compared a number of variables relating to the strength of TCR signaling. This was assessed independently by the degree of clonal expansion and resulting clonotypic composition, the degree of surface TCR downregulation, the activity of a Nur77-GFP reporter (Figure 8A) (31), and the degree of PD-1 and LAG3 expression. Although all these parameters can be directly affected by the strength of TCR signaling, their precise relationship or indeed their effect on Tfh differentiation is not necessarily linear.

For example, EF4.1 CD4^+^ T cells primed in WT hosts exhibited the strongest Nur77-GFP signals and highest degree of TCR downregulation, whereas those primed in *Rag2*^−/−^ hosts displayed the opposite phenotype (Figure 8B), indicating a direct correlation between Nur77-GFP intensity and TCR downregulation. However, EF4.1 CD4^+^ T cells primed in *Tcra*^−/−^ hosts downregulated their TCRs to the same degree as in WT hosts, but without the accompanying increase in Nur77-GFP reporter activity (Figure 8B). Also, partly discordant were the degree of TCR downregulation and Nur77-GFP reporter activity in Th functional subsets primed in the same host (Figures 8C,D). Th1 EF4.1 CD4^+^ T cells retained significant amounts of surface TCR, compared with Tfh or Th0 cells primed in WT hosts (Figures 8C,D), likely due to infrequent interaction of the former subset with B cells. However, Th0 cells exhibited significantly higher Nur77-GFP reporter activity, than either Th1 or Tfh cells in these hosts, despite comparable TCR downregulation with Tfh cells (Figures 7C,D). These results suggested that, although modulation of TCR signal strength is evidently different in EF4.1 CD4^+^ T cells primed in T cell-replete or T cell-deficient hosts, the effect of T cell lymphopenia on Th differentiation operated through additional mechanisms.

Given the complex patterns of correlation between independently measured variables and the degree of Tfh and Th1 differentiation, we next calculated a correlation matrix (Figure 8E). To this end, we assessed the relative contribution and possible interaction of 13 variables controlling Tfh differentiation measured in the 11 separate combinations of host and immunization or infection regimen described here. This analysis indicated three distinguishable clusters across all conditions, corresponding to each of the three major Th subsets observed in FV infection. Development of Tfh cells correlated most strongly with the activity of the Nur77-GFP reporter, taken to indicate the strength of signaling EF4.1 CD4^+^ T cells received, and also with the intensity of PD-1 expression (Figure 8E). In contrast, Th1 development exhibited strong anti-correlation with Nur77-GFP reporter activity and PD-1 expression levels and instead correlated with T cell lymphopenia and the availability of IL-2, together likely driving T cell clonal expansion (Figure 8E). Finally, uncommitted Th0 cells, although sharing many attributes with Tfh cells, appear to cluster separately, correlating strongly with LAG-3 expression, the degree of TCR downregulation and, the use of high-affinity TCRs (Figure 8E). Thus, our data suggest that Tfh differentiation is promoted by an optimal degree of TCR signaling, as well as by T cell competition.

## Discussion

It is now well recognized that both T cell-intrinsic and T cell-extrinsic factors shape the balance of CD4^+^ T cell differentiation into distinguishable functional subsets (1–8). However, the relative contribution of each of these factors in isolation or their potential intersection with each other in the context of diverse immunological challenges is still not fully understood. Here, we provided evidence to suggest that the CD4^+^ T cell response to a model retroviral antigen, presented during natural infection, is heavily skewed toward Tfh differentiation. This allowed us to identify the variables that best correlate with the degree of Tfh differentiation in this model and to accurately quantify their contribution.

The variable that exhibited the closest positive correlation with Tfh differentiation of env-reactive CD4^+^ T cells in all the conditions studied was the strength of TCR signaling, translating to increased transcription of the Nur77-GFP reporter. Also, directly correlating with both the degree of Tfh differentiation and Nur77-GFP reporter activity was the intensity of PD-1 expression. These findings are consistent with an instructive model, whereby stronger TCR signaling in Th cell precursors favors Tfh development, likely through stronger induction of the Tfh-promoting cytokine IL-21 (42, 48, 49).

Although stronger TCR signaling in Tfh cells was indicated by the increase in Nur77-GFP reporter activity and the intensity of PD-1 expression, it should be noted that not all correlates of TCR signaling followed a similar pattern. For example, the degree of surface TCR downregulation, generally proportional to TCR signal strength (50), did not correlate with the degree of Tfh differentiation. Moreover, expression of LAG3, which is also transiently induced by strong TCR signals (51, 52), showed no positive correlation with Tfh differentiation. Instead, the env-reactive CD4^+^ T cells with the highest TCR signal, assessed by both the activity of the Nur77-GFP reporter and the degree of surface TCR downregulation, appeared inhibited in their commitment to either the Th1 or Tfh subsets. These cells, which we refer to as uncommitted Th0 cells (44), also strongly correlated with co-expression of the inhibitory receptors PD-1 and LAG3.

Seemingly uncommitted Th0 cells are not typically observed in acute viral infections (44), but a similar population lacking either Th1 or Tfh characteristics has been described in CD4^+^ T cells primed during the chronic phase of LCMV Cl13 infection (18). In that model, the emergence of Th0 cells was linked to an initial defect in effector differentiation, indirectly caused by chronic IFN type I production (18). Eventually, CD4^+^ T cells primed during chronic LCMV-specific developed almost exclusively into Tfh cells (18).

The env-reactive CD4^+^ T cells with Th0 characteristics observed during FV infection are also closely related to Tfh cells, highlighting parallels between acute FV infection and chronic LCMV infection. However, as IFN type I production is not a prominent feature of acute FV infection (40), defective effector differentiation must have alternative explanations. Our results with the FV model suggest that TCR signaling above an optimal strength restrains differentiation of env-reactive CD4^+^ T cells, which would otherwise be committed to the Tfh subset. Indeed, in addition to displaying the highest Nur77-GFP reporter activity and preferentially expressed genes involved in active metabolism, Th0 cells were most noticeable in conditions associated with the highest antigenic load or potency. Furthermore, the frequency of Th0 in env-reactive CD4^+^ T cells was positively correlated with the percentage of PD-1 and LAG3 coexpressing cells across the various conditions studied and was reduced by anti-PD-1 and anti-LAG3 treatment during FV infection, which promoted their differentiation into Tfh cells. Notably, Tfh differentiation was also reported to be enhanced by anti-PD-1 and anti-LAG3 treatment during *Plasmodium yoelii* infection of mice (53), indicating that this pathway is restricting Tfh differentiation in persistent parasitic, as well as viral infection. Thus, our results suggest that Tfh differentiation is most efficiently induced by an optimal range of TCR signal strength.

Although evidently influenced by TCR signal strength, Tfh differentiation of the CD4^+^ T cell response to F-MLV env was independent of TCR clonotypic affinity, as previously suggested (37, 41, 42). Env-reactive CD4^+^ T cells bearing identical high-affinity clonotypic TCRs (identified by the use of endogenous Vα2 chains) were found both in the Tfh and Th1 subsets at comparable frequencies. Notably, despite comparable clonotypic TCR usage and Nur77-GFP reporter activity, Th1 env-reactive CD4^+^ T cells retained nearly the full amount of surface TCR, when compared with their Tfh counterparts in the same host, which had nearly lost their surface TCR expression. Therefore, the differential strength of TCR signals received by Th1 and Tfh cells cannot be solely attributed to differences in clonotypic TCR affinity. Instead, at least part of the differential TCR signaling between Th1 and Tfh cells is secondary to their differentiation and likely the result of their anatomic localization and interaction with distinct APC types. Supporting this notion, downregulation of surface TCR expression in CD4^+^ T cells has been previously shown, in FV infection (42), as well as in other model systems (54, 55), to be dependent on interaction with antigen-presenting B cells. Retention of surface TCR preferentially in Th1 cells would, therefore, suggest reduced B cell interaction. A central role for the APC type in determining or consolidating Th1 or Tfh effector differentiation may also underlie the propensity of different vaccine vectors or immunization regimens to induce distinct ratios of Th1 and Tfh response to a given antigen.

In addition to interaction with distinct APC types, our findings also support the concept that interaction and/or competition between T cells are also critical in determining effector differentiation, particularly, of Th1 cells. Although expectedly B cell deficiency did promote Th1 responses at the expense of Tfh responses, the strongest positive effect on Th1 differentiation was T cell lymphopenia. Indeed, the proportion of Th1 cells was approximately threefold higher in T cell deficiency than in B cell deficiency. Conditions conducive for Th1 differentiation in T cell lymphopenia are likely to involve deficiency in Treg cells, which can reduce availability of IL-2, thus promoting Tfh differentiation (24). However, an additional role for primary IL-2 production by effector CD4^+^ T cells was also indicated by single-cell RNA-sequencing analysis. Although Th1-promoting *Il2* transcription was still detected in single CD4^+^ T cells despite the heavy skewing toward a Tfh response following FV infection, this was always accompanied by transcription of Th1-suppressing cytokines, such as *Il21* and *Il10*. In contrast, *Il2* transcription in single CD4^+^ T cells primed by Ad5.pIX-gp70 vaccination partly overlapped only with *Gzmb* transcription.

Together, our results highlight the potent contribution of T cell-extrinsic variables to determine the relative balance of Th1 and Tfh responses. Manipulating these variables in vaccination regimens in order to achieve a balance of CD4^+^ T cell effector differentiation appropriate for the respective context (e.g., viral infection or cancer) will be the next important challenge.

## Ethics Statement

All animal experiments were approved by the ethical committee of the Francis Crick Institute, and conducted according to local guidelines and UK Home Office regulations under the Animals Scientific Procedures Act 1986 (ASPA).

## Author Contributions

LD and TD performed the experiments and analyzed the data. LD and GK wrote the manuscript. GK supervised the study and contributed to data analysis.

## Conflict of Interest Statement

The authors declare that the research was conducted in the absence of any commercial or financial relationships that could be construed as a potential conflict of interest.
